# Supplementary material for: Chromothripsis during telomere crisis is independent of NHEJ, and consistent with a replicative origin
Source: Genome Res. 2019 May;29(5):737–49. doi: 10.1101/gr.240705.118 (PMC6499312; doi:10.1101/gr.240705.118)
Supplement: Supplemental Material [file supp_gr.240705.118_Supplemental_file_1.zip › contigs/annotated_contigs/DB48/contig.2.DB48_length_319_mean_cov_2.09717868339.docx]

**DB48_length_319_mean_cov_2.09717868339**

```
ATAATAATAAAAAATAAAATAAAATAAAAT|AAA|GCATAGATGCTTTTTTTGTGAAGGAATTATAGAGCAAGGACATTTGGATTCATT
 >chrX:136571115-136571148 + E=2e-08 p=1e-02 >chr7:51672795-51673086 + E=2e-158
ATTTGAAATAATTAGTATTTGTTTTCTCTCCATCATGTCAAAACAGAGAATTTGAAGTAATTTCAAAACTAAGAGCAGTGGTCCAAAAG

AAAAAAAAAACTTCTCATAGTTTATCCATAGTTTAAACACAACAATCACTAATGAGCAGAACTGTGTCTATAAGATTTCTGGAGTTCAA

GGTGAGTAGGGAAACAAGTTTGGTAAGTCACAGTGCTGGAAAGGAGGAAATGGT
```
